# A case report of right coronary artery ligation in the treatment of adult - type anomalous origin of the right coronary artery from the pulmonary artery complicated with coronary heart disease

**DOI:** 10.3389/fcvm.2026.1804473

**Published:** 2026-04-29

**Authors:** Qiang Zhang, Hongyan Zhuo, Su Yan

**Affiliations:** 1 Department of Imaging, Yanbian Hospital, Jilin, China; 2 Cardiac Imaging Department, Jilin Central Hospital, Jilin, China

**Keywords:** anomalous origin of the right coronary artery from the pulmonary artery, case report, congenital heart disease, coronary artery atherosclerosis, coronary artery steal

## Abstract

Anomalous origin of the right coronary artery from the pulmonary artery (ARCAPA) is a rare congenital coronary artery malformation, with an adult incidence of approximately 0.002%. Due to the “coronary steal” phenomenon, it can lead to myocardial ischemia and pose a potentially fatal risk. This paper reports a case of a 54-year-old female with ARCAPA complicated by multi-vessel coronary atherosclerosis. Coronary CTA was used to clarify the malformed anatomy and the steal phenomenon, and to evaluate the degree of coronary artery stenosis. A staged treatment strategy was adopted: the main trunk of the right coronary artery was ligated first to eliminate the “coronary artery steal,” and intensive secondary prevention drug therapy for coronary heart disease was administered after the operation. The patient's symptoms were relieved, and follow-up showed improvement in cardiac function. This case highlights the value of coronary CTA in the diagnosis of complex heart diseases and demonstrates the importance of an individualized staged treatment strategy when congenital and acquired heart diseases coexist.

## Introduction

Anomalous origin of the right coronary artery from the pulmonary artery (ARCAPA) is a rare congenital coronary artery malformation, and the vast majority are isolated lesions (1). However, given the inherent bias in the group of patients undergoing surgery and the asymptomatic nature of some ARCAPA patients, it suggests that the true prevalence of adult ARCAPA may be underestimated (2). Due to the relatively low myocardial oxygen consumption in childhood, most patients have no obvious clinical symptoms and are often incidentally discovered in adulthood. Adult patients present with diverse clinical manifestations, which can range from being completely asymptomatic to presenting with angina pectoris, heart failure, arrhythmia, and even sudden cardiac death.

The core pathophysiological mechanism of this disease is the “coronary steal” phenomenon (3). Its hemodynamic driving mechanism lies in the pressure gradient change between the aorta and the pulmonary artery: during systole, the pressures of the aorta and the pulmonary artery are similar, and the degree of steal is relatively mild; however, during diastole, the aortic pressure remains at a relatively high level, while the pulmonary artery pressure drops rapidly, leading to a large amount of blood flowing reversely from the left coronary artery system to the lower - pressure right coronary artery through collateral circulation and finally flowing back into the pulmonary artery. This results in a reduction in effective myocardial perfusion, inducing myocardial ischemia, fibrosis, and cardiac insufficiency. If there is stenosis in the left coronary artery itself, the steal phenomenon can double the risk of myocardial ischemia (4).

This paper reports a rare case of a 54-year-old woman with anomalous origin of the right coronary artery from the pulmonary artery (ARCAPA) complicated by multi-vessel coronary atherosclerosis. The interaction between these two diseases increases the risk of myocardial ischemia and the complexity of treatment. This paper focuses on describing the diagnostic process, imaging evaluation, and the basis for formulating the staged treatment strategy, aiming to provide a reference for the clinical management of such complex cases.

## Case presentation

**Figure 1:**
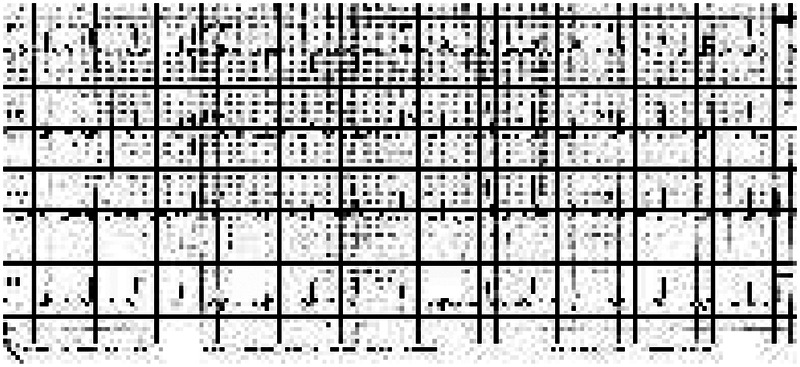
Preoperative electrocardiogram.

The patient, a 54-year-old woman, experienced palpitations and shortness of breath without obvious inducement while taking care of a toddler at home. She came to our hospital in August 2025, suspecting it was menopausal symptoms. The patient had a previous history of good health, with no history of hypertension, diabetes, angina, chest tightness, or syncope, and no family history of congenital heart disease. Physical examination revealed stable vital signs. A grade 2/6 soft systolic blowing murmur was audible at the left sternal border. The breath sounds of both lungs were clear, and there was no edema in both lower extremities. According to the New York Heart Association (NYHA) heart function classification criteria, the patient was rated as class II (Figure 1).

The initial electrocardiogram (ECG) showed sinus tachycardia (heart rate of 105 beats per minute), a prolonged QTc interval to 463 ms (normal value <460 ms), a normal electrical axis (QRS electrical axis of 37°), flattened and inverted T waves in the precordial and inferior wall leads; the leads Ⅱ, Ⅲ, and aVF presented a qR pattern, indicating a need to be vigilant for possible myocardial ischemia. To further determine whether there was myocardial injury, venous blood was collected for the detection of myocardial necrosis markers. The results showed that all were within the normal range, ruling out acute myocardial infarction. Subsequently, to further explore the cardiac structure and coronary artery conditions, a transthoracic echocardiogram was performed first, followed by coronary CT angiography.

**Figure 2:**
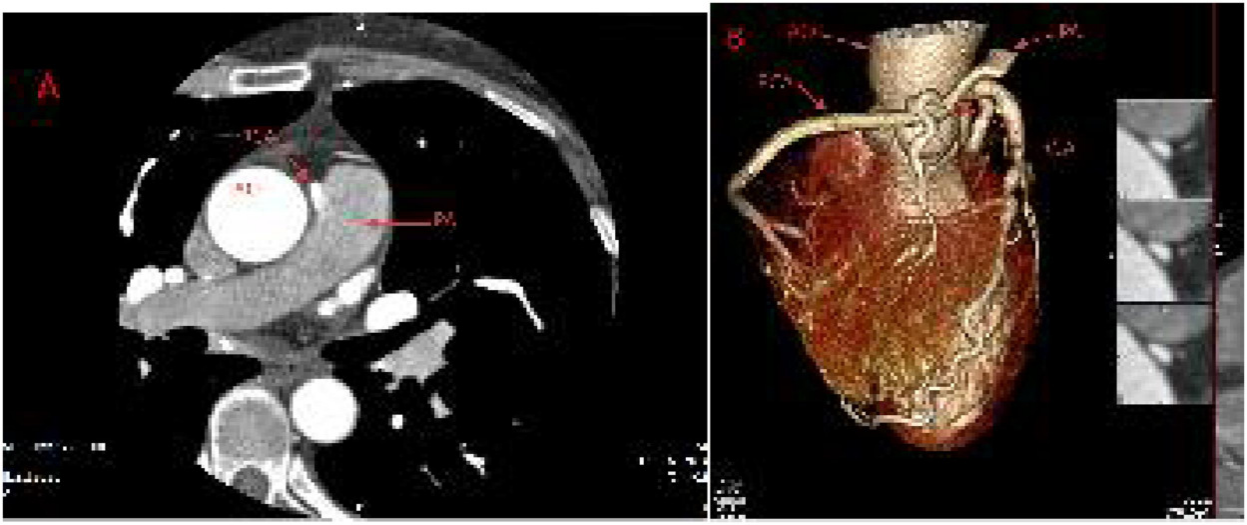
Preoperative coronary CTA. **(A)** Abnormal origin of the RCA from the pulmonary trunk (red arrow); **(B)** VR-reconstructed three-dimensional image shows the abnormal origin of the RCA (red arrow).

Transthoracic echocardiography showed that the left coronary artery (LCA) originated from the left coronary sinus, and the right coronary artery (RCA) originated from the main pulmonary artery. The left ventricular systolic function was normal, with an ejection fraction of approximately 67%. The left ventricular diastolic function was reduced (E/A < 1, e’ < a’) (Figure 2). Coronary CTA further confirmed that the RCA anomalously originated from the medial wall of the main pulmonary artery, approximately 32 mm away from the aortic valve. The distal ends of the left anterior descending artery (LAD) and the circumflex branch (LCX) of the left coronary artery were connected to the distal end of the RCA, forming a rich and tortuous collateral vascular network. During diastole, blood flow was observed to retrogradely perfuse from the LCA to the RCA through the collateral circulation and finally drain back into the pulmonary artery, presenting a typical “coronary steal” phenomenon. In addition, multiple calcified foci and eccentric non - calcified plaques were visible in the proximal segment of the anterior descending artery, with approximately 60%—70% luminal stenosis. No obvious abnormalities were found in the size of each cardiac chamber and the myocardial thickness.

Given that coronary CTA has clearly shown moderate stenosis (60%–70%) of the LAD and the patient has no obvious angina symptoms, after discussion by the cardiac team, it was decided not to perform invasive coronary angiography and functional assessments such as fractional flow reserve (FFR)/resting full-cycle ratio (RFR) for the time being. The main considerations are as follows: (1) The primary treatment goal for the patient is to eliminate the steal phenomenon and relieve the core cause of myocardial ischemia through right coronary artery ligation. (2) Given the moderate stenosis of the LAD and the patient's asymptomatic status, the hemodynamic significance is not yet clear. Since the collateral circulation mainly originates from the LAD, if FFR/RFR measurement is performed in the steal state, the true functional significance of the stenosis may be overestimated due to the abnormal hemodynamic state, which may mislead treatment decisions. Therefore, functional assessment should be conducted after the steal phenomenon is corrected and the myocardial blood supply state is stable. (3) The staged treatment strategy allows for re-evaluation of whether revascularization is needed for the left coronary artery lesions based on the symptoms and functional examination results after surgery.

**Figure 3:**
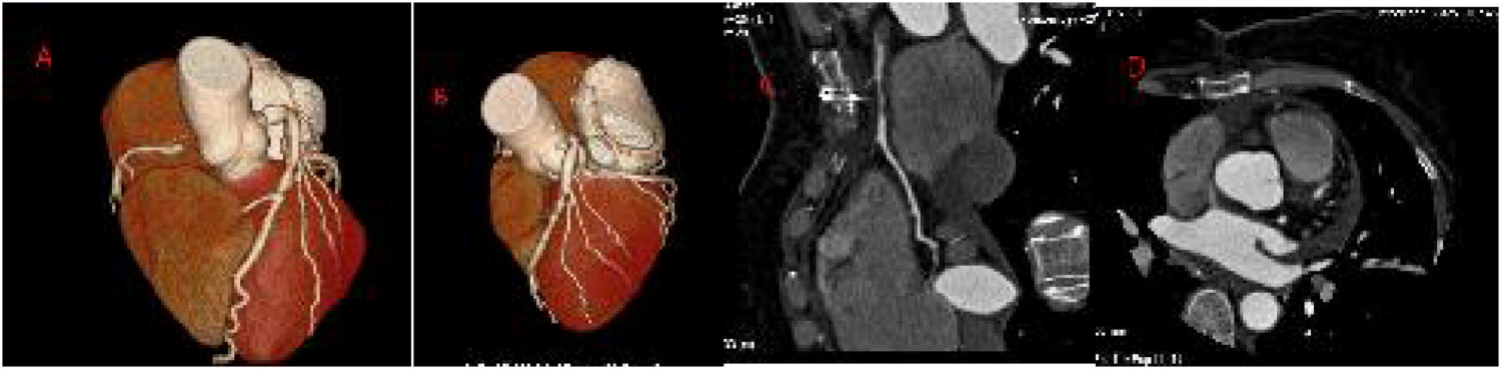
Coronary CTA at 2 months after surgery. VR-reconstructed three-dimensional images show the proximal occlusion of the right coronary artery and the collateral circulation network of LCA; CPR sagittal view shows the proximal occlusion of RCA; Axial four-chamber view shows the proximal occlusion of the RCA.

The patient underwent “ligation of the right coronary artery trunk” at a hospital in Shanghai on October 29, 2025. During the operation, exploration revealed that the RCA originated from the right side of the root of the main pulmonary artery, and the entire course was significantly thickened, with the diameter of the trunk being approximately 8 mm, and its distal end running within the aortopulmonary septum; the middle and distal segments of the LAD also showed thickening, with rich collateral circulation. A temporary occlusion test of the right coronary artery trunk was performed during the operation, and there were no significant changes in the patient's heart rate, blood pressure, and electrocardiogram, confirming that the blood supply area had completely relied on the collateral circulation from the LCA. On this basis, the right coronary artery trunk was doubly ligated with No. 7 and No. 4 silk sutures. The operation was smooth, with little intraoperative bleeding, and no blood transfusion was given. After the operation, the patient's symptoms were significantly relieved, and vital signs were stable. Perioperative secondary prevention drug treatment for coronary heart disease was initiated, with the specific plan as follows: dual antiplatelet therapy (aspirin+clopidogrel) for a planned duration of 6 months; then switched to long - term maintenance with aspirin. At the same time, statins were taken in combination for a long time, with the target LDL - C < 1.8 mmol/L, and dynamically adjusted according to lipid levels to stabilize plaques and delay the progression of atherosclerosis. The patient was discharged smoothly on the 5th day after the operation (Figure 3).

Two months after the operation, a coronary CT angiography (CTA) review showed the formation of a thrombus within the proximal lumen of the right coronary artery (RCA), with complete occlusion of the lumen. The abnormal blood flow signal disappeared, and the “coronary artery steal” phenomenon was corrected. The collateral circulation network between the left coronary artery (LCA) and the distal RCA was still visible, but the blood flow direction was stable without abnormal shunting. No RCA opening was still found in the right aortic sinus.

During the follow-up at 3 months after the operation, the patient's NYHA cardiac function class improved to Grade I. Clinical symptoms were significantly alleviated, and the patient reported no discomfort during daily activities. Echocardiography showed that the left ventricular systolic function remained normal (ejection fraction of 65%), and the left ventricular diastolic function improved compared to the preoperative state. The moderate - volume pericardial effusion present before the operation was also significantly absorbed. There were no dynamic ischemic changes on the electrocardiogram. These imaging findings collectively confirmed the dual success of right coronary artery ligation at both the anatomical and functional levels. Currently, the treatment focus has completely shifted to long - term pharmacological management of the patient's coronary atherosclerotic disease. According to the staged treatment strategy, a stress echocardiogram or myocardial SPECT functional examination is planned to be conducted 6 months after the operation to evaluate the hemodynamic significance of the moderate stenosis in the left anterior descending artery (LAD), and subsequent treatment directions will be determined accordingly. If the examination indicates inducible myocardial ischemia, invasive coronary angiography will be further performed. Revascularization, including percutaneous coronary intervention (PCI) or minimally invasive direct coronary artery bypass (MIDCAB)/conventional coronary artery bypass grafting (CABG), will be considered based on the characteristics of the lesions. If there is no definite evidence of ischemia, the pharmacological treatment will be continuously optimized, and regular follow - up will be carried out.

## Discussion

Anomalous origin of the right coronary artery from the pulmonary artery (ARCAPA) is a rare congenital coronary artery malformation in adults. In our hospital, only 1 case was detected among approximately 150,000 coronary CT angiography (CTA) examinations from 2010 to 2025. Most patients are asymptomatic for a long time due to the establishment of collateral circulation compensation and are often incidentally discovered during imaging examinations (5). The presence of the “coronary steal” phenomenon can increase the risk of myocardial infarction and sudden cardiac death. Therefore, once ARCAPA is diagnosed, surgical treatment is usually recommended to reduce the risk of adverse cardiovascular events (6). If a patient also has multi-vessel coronary atherosclerosis, the two diseases can interact at the pathophysiological level, making the clinical manifestations more complex (7). Treatment decisions also need to comprehensively consider the combined effects of anatomical abnormalities and myocardial ischemia.

Multidetector computed tomography coronary angiography (MDCT CTA), with its rapid data acquisition, high spatial resolution, and operational stability, has become the preferred method for evaluating coronary artery morphology (8). In this case, coronary CTA not only clearly demonstrated the anatomical structure of ARCAPA, fully presented its course and spatial relationship with surrounding tissues, but also dynamically showed the blood flow path of “coronary steal” and the collateral circulation network (9), and accurately evaluated the degree of coronary artery stenosis (10). It provided important anatomical and functional basis for formulating individualized treatment decisions.

Surgical operation is the core approach for radical treatment of ARCAPA and elimination of the “coronary steal” phenomenon. Direct reimplantation of the right coronary artery is the preferred surgical procedure for restoring physiological blood supply (11). However, for patients with severe coronary atherosclerosis, advanced age, left main coronary artery disease, or high risk of cerebrovascular events, direct reimplantation may be associated with relatively high peri - operative risks (12). Therefore, the “staged strategy” becomes an alternative approach: in the first stage, right coronary artery ligation can be performed first (13) to rapidly block abnormal blood flow and relieve myocardial ischemia; in the second stage, drug therapy or revascularization can be scheduled according to the patient's specific conditions (14). It is worth noting that invasive coronary physiology measurements (FFR/RFR) were considered before surgery in this disease to guide treatment decisions for LAD stenosis. However, performing such measurements in the state of coronary steal may overestimate the true functional significance of the stenosis. Therefore, it is a more reasonable decision - making path to conduct functional evaluation after the correction of the coronary steal phenomenon. In this case, preoperative evaluation and intraoperative temporary occlusion test confirmed the establishment of abundant collateral circulation, so right coronary artery ligation was selected. This surgical procedure not only eliminates the steal phenomenon but also avoids complex pulmonary artery anastomosis operations, creating favorable conditions for subsequent interventional or bypass treatments that may target LCA lesions. For this relatively young patient, if future functional evaluation indicates that LAD stenosis has hemodynamic significance, the choice of revascularization method needs to be comprehensively considered. Minimally invasive direct coronary artery bypass (MIDCAB) using the left internal mammary artery to bypass the LAD has the advantages of high long - term patency rate and less trauma, making it a very attractive option; while conventional CABG or PCI can also be used as alternative options, and the specific choice should be comprehensively determined by the cardiac team based on the patient's wishes and lesion characteristics. It should be emphasized that regardless of the surgical procedure used, reducing myocardial injury is crucial (15). Especially in patients with coronary atherosclerosis, measures such as optimizing anesthesia management and circulatory management and reasonably using myocardial protection drugs should be taken to minimize the secondary damage to the ischemic myocardium caused by surgery.

Postoperative management should shift towards long-term systematic prevention and treatment of coronary atherosclerosis. In this case, the patient has initiated a secondary prevention program centered on dual antiplatelet therapy and statins (16). For the functional significance assessment of moderate stenosis in the left anterior descending artery (LAD), a phased functional testing strategy was adopted in this case, which was carried out 6 months after the operation, aiming to clarify whether the LAD lesion still had hemodynamic significance after the correction of the steal phenomenon. The rationality of this strategy lies in: (1) avoiding invasive assessment before the steal phenomenon is corrected to prevent overestimation of the degree of ischemia; (2) the results of the functional test can provide an objective basis for subsequent revascularization decisions and avoid unnecessary interventional procedures; (3) it conforms to the principle recommended by the current guidelines for functional assessment of moderate stenosis lesions before deciding on revascularization (17). Whether further revascularization is needed in the future should be determined by a comprehensive evaluation of the cardiac team. Additionally, the patient already has left ventricular dysfunction, indicating long-term ischemic myocardial damage, and continuous monitoring of cardiac function changes is required. Although existing literature suggests that the overall prognosis after ARCAPA surgery is good (6), long-term follow-up data for patients with concurrent coronary heart disease are still insufficient. Therefore, it is recommended to establish a regular follow-up mechanism for postoperative patients, including coronary CTA evaluation of blood vessels 1 year after the operation, and subsequent imaging follow-up every 2–3 years; annual clinical evaluation and echocardiogram examination to monitor changes in cardiac function, myocardial ischemia, and arrhythmia for continuous observation of the patient's long-term prognosis; patients who have undergone reimplantation should also receive regular coronary CTA examinations to evaluate the patency of the anastomosis and the presence of complications such as aneurysms for continuous observation of the patient's long-term prognosis.

In conclusion, ARCAPA is a rare coronary artery anatomical anomaly with highly variable clinical manifestations. Currently, coronary angiography and CTA have become the main means for definite diagnosis. Although some patients have no obvious symptoms, considering the potential risks of myocardial ischemia and sudden death, surgical intervention is still regarded as the preferred treatment method, aiming to eliminate the “coronary steal” phenomenon and restore physiological coronary perfusion. This case shares the diagnosis and staged treatment process of a patient with ARCAPA complicated by multi - vessel coronary atherosclerosis, providing practical evidence for the standardized clinical management of this rare disease.

## Data Availability

The original contributions presented in the study are included in the article/supplementary material, further inquiries can be directed to the corresponding author.
